# Dyadic inter-group cooperation in shotgun hunting activities in a Congo Basin village

**DOI:** 10.1017/ehs.2024.14

**Published:** 2024-04-01

**Authors:** Vidrige H. Kandza, Haneul Jang, Francy Kiabiya Ntamboudila, Sheina Lew-Levy, Adam H. Boyette

**Affiliations:** 1 Department of Human Behavior, Ecology and Culture Max Planck Institute for Evolutionary Anthropology, Leipzig, Germany; 2 Institute for Advances Studies, Toulouse, France; 3 Faculte des Lettres, Arts et Sciences Humaines Marien Ngouabi University, Brazzaville, Republic of the Congo; 4 Department of Psychology Durham University, Durham, UK

**Keywords:** Inter-group cooperation, shotgun hunting, hunter–gatherers, Congo Basin, multi-level modeling, bushmeat

## Abstract

Understanding the dynamics of inter-group cooperation in human adaptation has been the subject of recent empirical and theoretical studies in evolutionary anthropology, beginning to fill gaps in our knowledge of how interactions across political, economic and social domains can – and often do – lead to stable, large-scale cooperation. Here we investigate dyadic intergroup cooperation in shotgun hunting in the Republic of the Congo. In the Congo Basin, inter-group cooperation between foragers and farmers is at the centre of an exchange system maintained by traditional norms and institutions such as fictive kinship. Here, we focused on what factors predict cooperative shotgun hunting exchanges between BaYaka and Yambe. We conducted structured interviews with 48 BaYaka hunters and 18 Yambe men who organise hunts in a village along the Motaba River. We used Bayesian multilevel regression models to investigate the influence of Yambe and BaYaka attributes on probability of dyadic cooperation. We found that BaYaka men's reputations as skilled hunters and their family size each predicted cooperation in shotgun hunting, whereas there was no effect of Yambe attributes (status, wealth, family size). We discuss the results in terms of evolutionary models of men as hunters and inter-group cooperation, as well as biodiversity conservation implications.

## Graphical abstract

**Figure:**
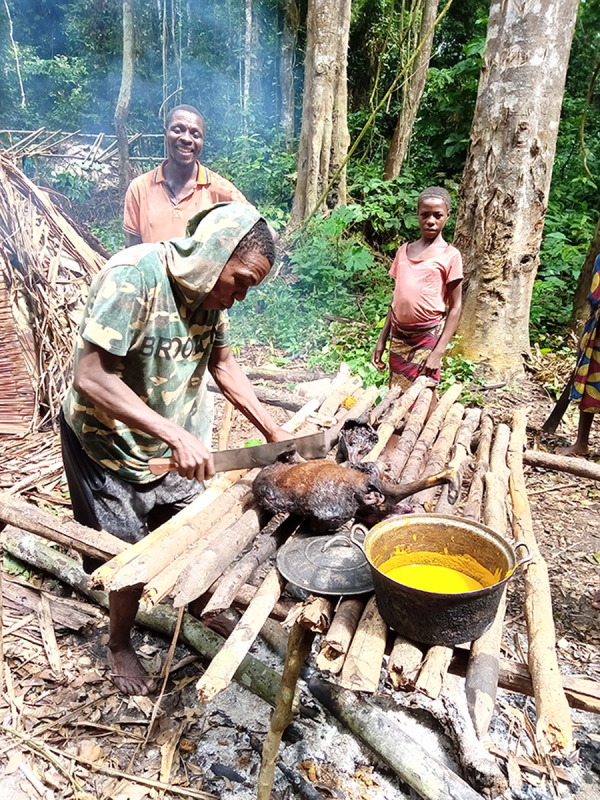

**Social media summary:** In a Congolese village, a hunter's skill and the needs of his family predict inter-group cooperation in shotgun hunting.

## Introduction

The human capacity to maintain relatively harmonious, interdependent relationships across group boundaries is one of the many ways in which cooperative behaviour is unique in our species (Barth, 1969; Boyd & Richerson, 2021; Robinson & Barker, 2017). Chimpanzees and bonobos, for instance, are intolerant towards neighbours, and interactions tend to be aggressive (Glowacki, 2022; Pisor & Surbeck, 2019; Wrangham & Glowacki, 2012). While some observations of the latter species suggests that scope for inter-group cooperation may be phylogenetically deep in our evolutionary lineage (Lucchesi et al., 2021; Pusey, 2022), the scale, duration and diversity of cooperation between human groups is unprecedented. For humans, seeking out and maintaining inter-group cooperation was critical for the evolution of culture, and probably played an important role in human migration and cultural adaptation across time (Boyette et al., 2022; Fuentes, 2004; Miller & Wang, 2022). One important way groups stabilise cooperative relationships against conflicts is through the exchange of resources that each group is more efficient at acquiring (Robinson & Barker, 2017). Such a division of labour spreads risk across the socio-ecological landscape so that cooperating groups can receive the benefits of mutualism and effectively buffer against resource shortfalls in dynamic environments (Cashdan, 1987, 2001; Colson, 1979; Migliano et al., 2017; Pisor & Surbeck, 2019; Wiessner, 1982). Moreover, while stabilised by the economic benefits of trade and resource diversification, inter-group ties also serve as pathways for cultural exchange, leading to the spread and production of cultural diversity, as groups adopt and modify knowledge, beliefs and practices from their cooperative partners (Ben-Oren et al., 2023; Sterelny, 2020). Thus, understanding the economic, social and cultural mechanisms through which individuals and groups maintain inter-group cooperation is critical to a full understanding of the dynamics of human cooperative behaviour and cultural evolution.

In this paper, we focus on a particular instance of inter-group cooperation rooted in economic specialisation and exchange. Specifically, throughout the Congo Basin of central Africa, Bantu-speaking peoples specialised in swidden–horticultural practices maintain institutions of exchange with those peoples who are specialised in hunting and gathering in this complex tropical forest ecology (Bahuchet & Guillaume, 1982; Köhler & Lewis, 2001; Takeuchi, 2014; Terashima, 1986). Historically, the migration of the Bantu-speakers into this region (hereafter ‘farmers’) was probably possible because of the early establishment of trade relations with the ancestors of the current Indigenous forest peoples (hereafter ‘foragers’), around 5000 years ago (Klieman, 2003; Patin & Quintana-Murci, 2018). Ethnographically, contemporary farmers attribute their ability to live in the forest ecology to the help of the foragers, and the foragers attribute their knowledge of agriculture and cultigens to their ancestors’ relations with those of today's farmers. Since its inception, this system of exchange became widespread and has remained stable at the regional scale, while remaining highly dynamic at more local levels (Boyette et al., 2022).

Archaeologists have long been interested in the transition of foraging populations to farming populations, but there is less research on the intervening periods of dynamic interaction between these two groups which, in many cases, lasted millennia (Juwayeyi, 2011; Spielmann, 1986; Spielmann & Eder, 1994). Although there have been major debates over how to define forager–farmer relationships in general terms (Fortier, 2001; Hart & Hart, 1986; Headland, 1987; Köhler & Lewis, 2001; Solway & Lee, 1990; Wilmsen & Denbow, 1990; Yasuoka, 2013), the specific social, cultural and economic factors which supported or hindered inter-group cooperation between individual foragers and farmers remains understudied. Yet the dynamics of these dyadic relationships may provide insights into much larger patterns of population-level stability and change (Pisor & Gurven, 2018), and to analogous systems of ‘forager–farmer exchange’ described elsewhere (e.g. Bird-David, 1990; Endicott, 1984; Fortier, 2001; Griffin & Griffin, 1997).

Focusing narrowly on the Congo Basin and even more narrowly on the forests of northern the Republic of the Congo, forager–farmer relationships remain the major pathways for the movement of knowledge and resources from the forest to the world outside (J. Lewis, 2005). Through the BaYaka people's procurement of forest resources for their own use and for trade, both the forager and farmer communities depend on the forest for their subsistence. Moreover, many forest resources gathered by foragers are sold nationwide, including foods, medicines and other materials like *mongongo* leaves that are used to wrap the ubiquitous *kwanga* manioc bread, making these relationships foundational to an unknown proportion of the Congolese economy. Importantly, the region is also being actively logged by international forestry companies whose workers are fed by bushmeat that comes in part from sale by farmers who acquired it through exchange with the foragers (Kandza et al., 2023). Thus, there is some urgency to understanding how these exchange relationships in general, and those around hunting in particular, impact the management of biodiversity in this region, a global biodiversity hotspot (S. L. Lewis, 2006).

In sum, because of the potential of this study system to inform our wider understanding of forager–farmer relationships and because of its particular significance to the specific ethnographic context, this study examines which traits of individual BaYaka forager and Yambe farmer men predict their dyadic cooperation in shotgun hunting exchange. Importantly, we do not make the case that these inter-group cooperative relationships are always peaceful (Glowacki, 2022). In fact, conflicts between individual BaYaka and their Yambe exchange partners are common. Animosity, ethnocentric attitudes and outright disdain are at times present in both groups towards the other (J. Lewis, 2005). So are friendship, loyalty and mutual respect (Rupp, 2011). Our interest here is to identify some of the ethnographically grounded parameters that tend to support the dyadic relationships that themselves constitute the region-wide institution of Congo Basin forager–farmer exchange, and to describe the extent to which these parameters are theoretically generalisable beyond this case study.

## Intergroup cooperation in shotgun hunting

In the study village, Yambe farmers have longstanding traditional exchange relationships with the BaYaka foragers who live in association with their village. Many local BaYaka families move between their village residences and forest settlements to harvest different resources throughout the year (e.g. caterpillars, fish), but individuals may go into the forest every day to harvest specific resources even when they are settled in the village. While near the village, their forest activities are often for the purpose of exchange or sale to the Yambe. Yambe will call upon the BaYaka to collect a variety of forest resources, including palm nuts, palm wine, several species of nut, black pepper, *Gnetum* leaves and game meat – the subject of this study. Yambe also will recruit BaYaka labour for household tasks or for work in their horticultural gardens. What BaYaka receive in exchange for their goods or labour varies, but in the study village, BaYaka most often receive agricultural produce, commercial goods or alcohol for their work. Cash is rarely given as compensation in the study village.

Exchange relationships between the BaYaka and the Yambe share features that serve to stabilise intergroup cooperation more generally (Fearon & Laitin, 1996; Robinson & Barker, 2017; Wiessner, 1982). As is common throughout the Congo Basin, intergroup cooperation in the study village is organised by an institution of fictive kinship (Joiris, 2003). Fictive kinship represents a form of social ties not based on either consanguineal or affinal kinship. Rather, fictive kinship connects individual foragers to individual farmers who call each ‘family’, and these linkages between individuals extend to their consanguineal families and are inherited across generations as well as acquired through marriage (Joiris, 2003). These fictive kinship relations organise the exchange of goods, labour and specific services such as divining or conducting ceremonies (J. Lewis, 2002; Takeuchi, 2014). Our work among the BaYaka and Bantu-speaking peoples in the Motaba River region of the Republic of the Congo, where the current study was conducted, has also shown that inter-group cooperation is further stabilised by shared norms of exchange, despite otherwise contrasting cultural norms regarding cooperation and resource sharing, and individual differences in goals and obligations (Jang & Boyette, 2021; Kandza et al., 2023; Pope-Caldwell et al., 2022).

In shotgun hunting exchanges specifically, a set of shared norms govern the organisation of hunts and the compensation to hunters. The critical feature of these exchanges is that the Yambe own the shotguns, but the BaYaka have the skill and traditional ecological knowledge to successfully hunt. As described more fully by Kandza et al. (2023), the nature of the exchange varies depending on the quantity of game the organiser of the hunt is hoping to receive. Yambe will recruit BaYaka to hunt game for their families’ immediate consumption but also to sell on the bushmeat market. The money they earn is used to support their children's school or buy medicine, clothes or other needs (Kandza et al., 2023). Without direct access to markets or schools, BaYaka hunters claim to participate in these exchanges only for meat to share with their family and other group members (Kandza et al., 2023). While this may appear to give asymmetrical power to the Yambe, who have greater control over the flow of cash, it is important to note that hunters can refuse to hunt for the Yambe, and state that they choose to pursue this type of hunting because of the efficacy of the shotgun (Kandza et al., 2023). Moreover, in other parts of the northwestern Congo Basin, it has been shown that foragers continue to trade meat with farmers even when they can sell meat for cash, probably because of the value of maintaining the social relationship with the farmers (Lupo & Schmitt, 2017). Further, as the normative public payment for these hunts is only the head and organ meat from each animal, in addition to some quantity of cigarettes and alcohol, hunters often take advantage of the inability of the Yambe to police their activities in the forest, and will keep entire kills for themselves, often larger species than those they return to the organiser (Kandza et al., 2023). As the Yambe can return a significant profit through bushmeat sales, they accept this risk. Thus, for both groups, the practice is valued and participation is high (Kandza et al, 2023).

In this paper, building on Kandza et al. (2023), we aim to investigate which individual-level factors explain the variation in dyadic cooperation in shotgun hunting exchanges. Previously, we found that all hunters were fictive kin of their Yambe cooperation partners. However, the flexibility of the fictive kinship system gives the BaYaka as well as Yambe choices to select with whom to cooperate among a range of potential exchange partners, and there is individual variation in degree of participation in shotgun hunting exchanges. For instance, Yambe hunt-organisers mentioned working with a range of between one and 15 BaYaka hunters. Similarly, BaYaka named between one and nine specific Yambe as cooperative partners (Kandza et al., 2023). Below, we test theoretically and empirically grounded hypotheses and two ethnographically derived exploratory questions regarding why specific dyads cooperate in shotgun hunting exchanges in the study village.

### Hypothesis 1 (H1): hunter skill

More skilled hunters have been shown to be preferred cooperative partners in a variety of ethnographic contexts (E. A. Smith, 2004; K. M. Smith & Apicella, 2020). For BaYaka men, hunting is a major source of pride and identity. BaYaka men are generally famous for their hunting prowess, but skill and experience vary between individuals, and hunters’ reputations are well known in BaYaka and Yambe communities for several reasons. For both groups, meat plays a central role in the diet and there is a strong preference for game meat specifically (Dounias & Ichikawa, 2017; Kandza et al., 2023). For the BaYaka, men's reputations for hunting may also signal additional personal qualities that make them valuable cooperation partners or, for women, potential mates, as is the case in other hunting societies (E. A. Smith, 2004; K. M. Smith & Apicella, 2020). For the Yambe, information on BaYaka hunters’ skill is valuable because of the relative expense of buying shotgun cartridges and the possibility of significant profit from the sale of bushmeat from their recruitment of hunters from their BaYaka fictive kin (Kandza et al, 2023). Thus, we expect that Yambe are attuned to the reputations of the hunters with whom they choose to collaborate, and that these reputations accurately reflect their relative skill. Therefore, we first predict that the BaYaka with reputations for good hunting skills will preferentially be recruited to hunt and are thus more likely to have a greater number of Yambe cooperative partners.

### H2: BaYaka need and H3: Yambe need

In subsistence societies, game meat provides a critical source of complementary nutrients to garden-produced and other forest-procured foods (Gurven, 2004; Pontzer & Wood, 2021; Reyes-García et al., 2018). In many diets, it can be a critical source of protein, as well as minerals and vitamins such as iron, zinc, selenium and vitamins A, B6, B12 and E (Crittenden & Schnorr, 2017; Godfray et al., 2018). Given its value for supporting growth and development, meat can be particularly important in high-fertility contexts, including in the current case study (Headey et al., 2018; Krebs et al., 2011; Wyness, 2016). Hunts are by far the largest source of meat consumed in the study village and game is preferred over domestic animals (e.g. goats and sheep), which are primarily kept for sale, not consumption. ‘Meat hunger’ – specifically referring to a craving for wild meat – is documented as a distinct experience in the Congo Basin as well (Dounias & Ichikawa, 2017), and BaYaka fathers, specifically, are expected to bring game meat to their families as part of their culturally defined role (Boyette et al., 2020). Lastly, it is agreed upon by both groups that shotgun hunting exchanges are the most efficient way to harvest game (Kandza et al., 2023). Thus, for both BaYaka (H2) and Yambe (H3), we expect that individuals who have larger families are more likely to have a greater number of cooperative partners.

### H4: Yambe status

Cross-cultural research has shown that high status individuals tend to leverage their status to acquire a range of social benefits, including more cooperation partners (Garfield et al., 2020; C. von Rueden et al., 2008; C. R. von Rueden & Jaeggi, 2016). Thus, for the Yambe, social status may affect people's ability to recruit hunters from among their BaYaka fictive kin. In general, Yambe social organisation is based upon relative status. While age and male gender confer status, people also acquire status through demonstrating deep traditional knowledge of village family histories, through taking responsibility over village affairs (e.g. resolving conflicts, helping others) and through success at diverse economic activities. High status Yambe are also often generous to the BaYaka, even outside of the contexts of exchange (e.g. sharing food or basic items such as salt and soap), which can add to their reputation for generosity and kindness. Thus, in the context of hunting exchanges, high status Yambe may be prioritised by BaYaka hunters as cooperative partners. Therefore, we predict that Yambe who have higher status may have more BaYaka cooperative partners.

### Exploratory question 1 (E1): Yambe wealth

Among the Yambe, while wealth can lead to status, it can also be an independent pathway to power. Organising hunts is one of many Yambe economic strategies that require investments of cash (e.g. supplies for palm wine cultivation, *poivre noire* harvesting; Kandza et al. 2023). As such, wealthier people may be more likely to buy rifles and cartridges and thus to hire more BaYaka hunters, independent of their status (e.g. for generosity; see H4 above). Wealth as a means of power independent from status is relatively new to the village, reflecting new market intersections (e.g. bushmeat trade). Thus, we explore the independent role of wealth in cooperation in shotgun hunts.

### E2: Yambe fictive kin inheritance

Yambe are patrilineal and patrilocal, meaning that BaYaka fictive kinship is constant between generations of men. Additionally, various degrees of kinship between members of the Yambe community can also potentially be claimed to fit the norm of exchange through fictive kinship. However, because fictive kinship is also inherited through mothers and through marriage, we will test the prediction that a Yambe may increase the probability of having more cooperative partners if either their mother (E2a: Fictive kinship inheritance through maternal inheritance) or their spouse (E2b: Fictive kinship inheritance through marriage) is from the village.

### Adjustment variables

Finally, for both Yambe and BaYaka, we will adjust the statistical models used to examine the above predictions for the potential role of age as a covariate in predicting cooperation. Hunting skill, number of children, status, wealth and likelihood of marriage are each correlated with age, so the models must rule out this potential confound. We also make no specific prediction regarding the relationship between age and maternal natal location; however we adjust for age to rule out an unexpected relationship. We recognise that a man's age cannot cause his mother's natal location, but it is possible that a cohort of men of similar age could have fewer cooperative partners if their mothers had moved to the village during a single migration event. Thus, the model accounts for this possibility. Similarly, we do not make specific predictions regarding the direct effects of either Yambe or BaYaka men's age on their likelihood of cooperation, but the model adjusts for the potential of such effects. Direct cohort effects are possible if for unmeasured reasons more men of a particular age have more fictive kin or have a greater interest in shotgun hunting (e.g. younger men with more interest in markets). Alternatively, there is the possibility that older men in both groups have more experience cooperating with their fictive kin from the other group and are therefore better able to maintain more of these partnerships.

## Methods

### Ethics and consent procedures

Permissions to conduct research in the Republic of the Congo were obtained from the Institut de Recherche en Sciences Exactes et Naturelles and the Comité d'Éthique of the Institut National de Recherche en Sciences Sociales et Humaines in Brazzaville. The consent of the community was obtained after a public meeting in which the research aims and methods were explained. Individual consent of all study participants was then obtained one-on-one. All participants agreed to participate in the surveys within the framework of this study.

### Data collection

The study was conducted between May–July 2021 and May–June 2022 in a village located on the banks of the Motaba River and on the outskirts of the Nouabale Ndoki National Park in the large forest area occupied by the Congolese Industriel du Bois logging company in the Likouala Department in the Republic of Congo. Two ethnic groups, the BaYaka foragers and the Yambe fisher–farmers, have lived together for generations in this village. The cohabitation between these two ethnic groups is maintained and sustained at the individual and family level through multiple social relationships, such as fictive kinship ties and economic activities (e.g. exchange of food products, work tools; see Kandza et al., 2023 for detailed description of the ethnographic setting). We conducted interviews using a semi-structured questionnaire with a sample of BaYaka hunters (*n* = 48) and a sample of Yambe hunt organisers (*n* = 18) in the village. All interviewees were males. All Yambe households had guns or access to guns through familial relationships with other households, so there is no variation in the community in terms of ability to participate in shotgun hunting exchanges. Individuals from each ethnic group were approached by the interviewers on the way to the field or the river, or at home, depending on the availability of participants. The different sets of questions asked of hunters and hunt organisers can be found in Table S1. All interviews were conducted between 07:00 and 17:00. Interviews with Yambe participants were conducted by the first author directly in Lingala. For the interview with the BaYaka hunters, we hired a BaYaka translator who speaks both Lingala and Yaka. Each interview took less than 30 minutes. Among the 18 Yambe, 17 were married. The unmarried man lived at his father's house, while the rest of the Yambe men lived in their own house. All 48 BaYaka hunters were married, living in their own house with their family.

### Model variables

#### Yambe–BaYaka cooperative dyad

Our outcome variable is the presence of a cooperative exchange between a Yambe and a BaYaka hunter. For this, we asked Yambe in our sample to list the names of all BaYaka hunters with whom they cooperate for shotgun hunting, as the Yambe propose the hunts. From these data, we created all possible dyads between our sample of Yambe (*N* = 18) and our sample of BaYaka (*N* = 48). If they cooperated in shotgun hunting, we recorded their dyadic relationship as 1, and if not, 0. In total, we had 115 cooperative relationships and 749 cases of non-cooperation among 864 all possible dyadic relationships.

#### Yambe and BaYaka genetic kinship

To account for whether close genetic relatives among the Yambe and BaYaka, respectively, are more likely to cooperate with the same people from the other group (i.e. by virtue of sharing the same fictive kin), we included a kinship matrix for each Yambe and BaYaka, respectively, in the model (Hadfield & Nakagawa, 2010). These matrices incorporate the relative degree of genetic kinship between all Yambe and between all BaYaka who are involved in the study. In the kinship matrix, covariance is proportional to the coefficient of relatedness (*r*).

#### Skilled hunter nomination

To examine hunting skill among BaYaka hunters in the village, we used a free listing approach by asking hunters (*n* = 48) to rank the names of hunters who are good at hunting. When the interviewee stopped naming, the interviewers asked ‘Who else is a skilled hunter?’ until the interviewee said ‘I have named all the skilled hunters I know’. Twenty-two among 48 hunters were nominated by peers at least once, and the remaining 26 hunters were not nominated by anyone. We then computed a salience index for each hunter using the method outlined by Smith (J. J. Smith, 1993). This ‘Smith's *S*’ index accounts for the mean rank and the sum rank based on the number of lists generated. We used the men's reputation among their peers (e.g. as opposed to among the Yambe) as we assumed this would be the most accurate assessment of relative hunting ability.

#### Number of children

We asked BaYaka and Yambe participants how many living biological children they have. The mean number of children for participating Yambe was 6 (standard deviation, SD = 4.14, range 0–14 children) and the mean number of children for BaYaka was 3.6 (SD = 2.25, range 0–9 children).

#### Yambe status

We used membership on the village council as a proxy for Yambe status. Membership on the council is reserved for those with deep knowledge of village life and who can effectively solve community problems. Council members are invited by the appointed village chief, who consults with the oldest men in the village, who themselves have a place on the council by tradition. To record this variable, we asked Yambe participants whether they were members of the village council. If they were members, we recorded as 1, and if not, 0. Ten among 18 Yambe in the sample were on the council.

#### Yambe wealth

We used two proxies for wealth among the Yambe: ownership of a sheet metal roof on their dwelling and the number of shotguns owned. Sheet metal roofs are expensive and must be transported by motorised vehicle from the town to the village, and therefore reflect the relative wealth of these households compared with those with the more common thatched roof woven from the leaves of a palm gathered in the forest. We asked the participating Yambe whether they had a metal roof on their house in the village. If an interviewee owned a metal roof, we recorded as 1, and if not, 0. Ten among 18 had metal roofs. Shotguns are expensive as well and are therefore also a direct measure of relative wealth. While all Yambe sampled organised shotgun hunts, some had to borrow or rent the shotgun from others. Yambe were asked how many guns they personally owned and this number was recorded. Seven participants did not own a gun, five owned one and six owned two. No Yambe had more than two guns.

#### Yambe mother's and wife's natal locations

We asked Yambe men in which village their mothers and their wives were born. If the mother's natal village was the same as the current resident village of the Yambe participant (our study village), we recorded it as 1, and if the mother was born in another village, we recorded it as 0. The mothers of sex among 18 Yambe were not from the study village. Similarly, if a man's wife's natal village was the same as the current resident village, we recorded it as 1, and if the wife was born in another village, we recorded it as 0. Two among 18 men had two wives. In both of these cases, one wife was from the study village and the other was from a neighbouring village. These cases were coded as 1 to reflect the man having at least one chance to inherit BaYaka fictive kin. The wives of 12 among 18 Yambe men were born in other villages. The unmarried man was coded as having no data for this variable and his data was not used for statistical analyses.

#### Age

Yambe possessed ID cards with accurate birth year information, and these years were used to calculate their age. Since this is not the case for the BaYaka hunters, we asked the Yambe fictive kin of the hunter to estimate the hunter's age. The Yambe used their knowledge of the relative ages of the hunters in comparison with Yambe who were born around the same time. The mean age of the Yambe men was 46 years (SD = 9.62, range 33–64 years) and the mean (estimated) age of the BaYaka hunters was 32.5 years (SD = 10.05, range 20–55 years).

### Statistical analyses

We used Bayesian multilevel regression models in the Stan computational framework (http://mc-stan.org/), accessed with the function ‘brm’ of the brms package v. 2.16.3 in R v. 4.1.0. The unit of analysis in the statistical models was the 864 possible dyadic relationships in shotgun hunting activities between the 48 BaYaka hunters and 18 Yambe. As noted above, the response variable was binary: either a Yambe and a BaYaka cooperate (yes) or not (no) in shotgun hunting activities. In total, we had 115 cooperative relationships among all of the 864 possible dyadic relationships (115 yes and 749 no). To examine variation across individuals, we first fitted one baseline model, which accounts for the multilevel structure of data but includes only random effects for Yambe ID and BaYaka ID, Yambe kinship matrix and BaYaka kinship matrix, and no fixed effects. This approach accounts for the relative role of individual variation and genetic relatedness in determining the likelihood of participating in shotgun hunting exchanges among both the BaYaka and Yambe participants. We checked the standard deviations of the log-odds for cooperation between individuals and calculated intra-class correlation coefficients to investigate the proportion of variance captured by each random effect level of Yambe ID, BaYaka ID, Yambe kinship matrix and BaYaka kinship matrix (Nakagawa et al., 2017). We then fitted five main models including individual-level predictors and these four random effects. All models were fitted with a binary distribution.

**Figure 1.**
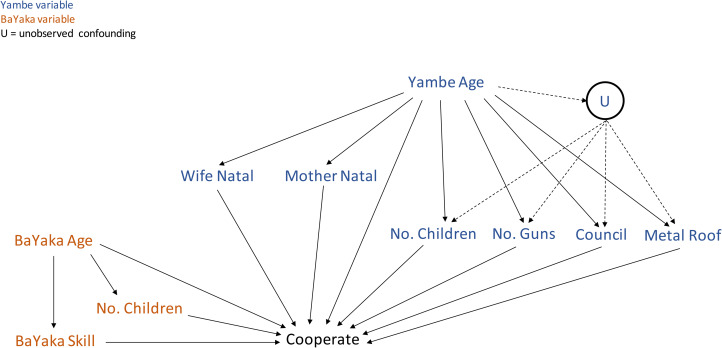
Directed acyclic graph (DAG) for our statistical models. This DAG illustrates the proposed causal associations between the main factors investigated in the study. Each variable is examined as a cause of cooperation, but both BaYaka and Yambe age are expected to influence cooperation through their effects on the main predictor variables. The unobserved confound ‘U’ reflects potential unmeasured variables that may mediate the effects of age on number of children, status (council) and wealth (metal roof, number of guns).

Among the five main models, two models investigated BaYaka attributes, specifically BaYaka's age, their nomination as a skilled hunter (H1) and their number of children (H2); and another three models investigated how Yambe attributes, including Yambe's age, the number of children (H3), council membership (H4), owning a metal roof or one or two shotguns (E1), and mother and wife's natal location (E2), could predict a cooperative relationship between a BaYaka hunter and a Yambe. These models are summarised in Table 1. We first represented the assumed causal links between the variables of interest using a directed acyclic graph (DAG), which helps us decide which variables should be included in the model to limit confounding, depending on our causal model (Figure 1). To do this, we used the ‘adjustmentSets’ function in the dagitty package in R. The results from ‘adjustmentSets’ function indicated that we should either adjust for Yambe's age + U (unobserved confounder) or adjust for Yambe's age + council + metal roof + the number of guns that the Yambe owned. Both adjustment sets are valid for recovering an unbiased estimate; however, only the second is possible because U is, by definition, unobserved. Hence, the first BaYaka model included BaYaka's age and BaYakas’ hunting skill nomination, and the second BaYaka model included BaYaka's age and the number of children as covariates, with the same random effects from the baseline model. The first Yambe model included Yambe's age, the number of children, council membership, having a metal roof and the number of guns as covariates, and the four random effects from the baseline model. The second Yambe model included Yambe's age and mother's natal location, and the third Yambe model included Yambe's age and wife's natal location as covariates. The results from the ‘adjustmentSets’ function indicated a need to adjust for BaYaka age.

**Table 1.** Model summary

Model	Predictors variables	Relevant hypothesis or exploratory question
1. BaYaka skills	Age + hunting skills	H1
2. BaYaka need	Age + number of children	H2
3. Yambe need, status and wealth	Age + number of children + council membership + metal roof + number of guns	H3, H4, E1
4. Yambe maternal inheritance of ties	Age + mother natal location	E2a
5. Yambe marriage inheritance of ties	Age + wife natal location	E2b

All models contain random effects for BaYaka and Yambe ID and genetic relatedness within the sample. Age, Age in years (estimated for the BaYaka – see text); wife's natal location – 1 if Yambe man's wife was born in the study village, 0 if not; mother's natal location – 1 if Yambe man's mother was born in the study village, 0 if not; number of children, total number of man's living biological children; hunting skills, ‘Smith's *S*’ index of salience; status, 1 if the Yambe man was a member of village council, 0 if not; metal roof, 1 if the Yambe man had a metal roof on his dwelling, 0 if not; number of guns, the number of guns own by the Yambe man.

All quantitative predictors were standardised to a mean of zero and standard deviation of one before fitting the models. We used weakly informative normal priors to guard against overfitting. We obtained posterior distributions of the effects of predictors from four independent Markov Chain Monte Carlo chains each with 4000 warmup and 2000 sampling iterations. We compared each of the five main models with the baseline model with information criteria, using model weights in the brms package. We found that even though there is high uncertainty in these effects, the models with covariates were favoured by model comparison.

## Results

In the baseline model including only random effects for Yambe ID, BaYaka ID, Yambe kinship and BaYaka kinship, we found that there is substantial variance in probabilities of participating in shot-gun hunting among Yambe individuals as well as among BaYaka individuals, which means that some individuals tend to cooperate more than others. These individual differences were larger in BaYaka hunters than in Yambe, which may be explained by the fact that all Yambe in our sample cooperated with the BaYaka hunters, while not all BaYaka cooperated with Yambe.

**Figure 2.**
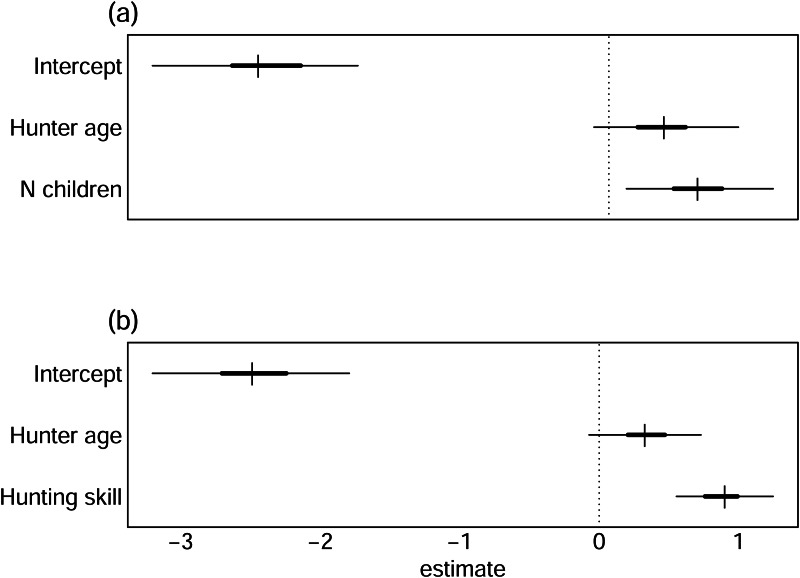
Forest plots for BaYaka hunter attributes. These plots show estimated of changes in probabilities of BaYaka hunters participating in cooperative hunting with Yambe hunt organisers, depending on (a) the number of living biological children of the hunters and (b) hunting skill, accounting for hunter's age. The point estimate is the median. The outer and inner lines represent 50 and 95% credibility intervals, respectively.

**Figure 3.**
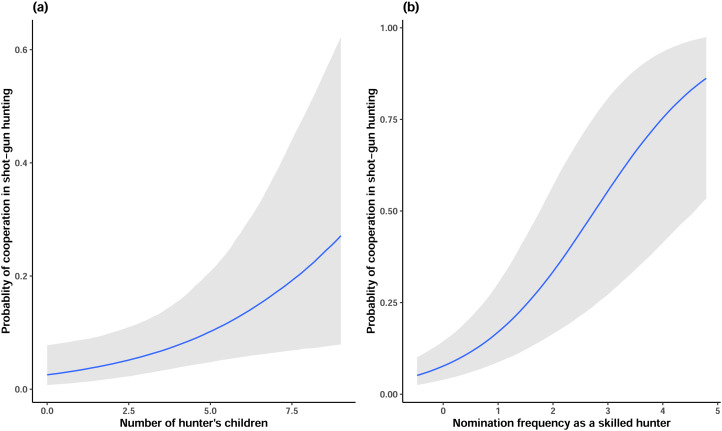
Posterior probability estimates of (a) hunter's number of living biological children and (b) skill as a hunter on their likelihood of cooperation in shotgun hunting.

While accounting for these individual differences, we found considerable effects of BaYaka hunting skill (Model 1) and hunters’ number of children (Model 2) when controlling for their age, in support of H1 and H2 (Figure 2). When BaYaka hunters had more children, they were more likely to cooperate in shotgun hunting with Yambe compared with when they had a lower number of children (Figure 3a). Moreover, when BaYaka hunters were nominated more frequently as a skilled hunter by peers, the probability of cooperation in shotgun hunting with Yambe was higher (Figure 3b).

None of the predictors in the models with Yambe attributes (Models 3–5) indicated effects on the likelihood of a cooperative relationship between a BaYaka hunter and a Yambe. Thus, no support was found for an effect of Yambe need (H3) or social status (H4), nor for any effects of Yambe wealth (E1) or fictive kin inheritance, either through mothers (E2a) or marriage (E2b) (Figure S2).

## Discussion

In this study, we tested several predicted relationships between individual attributes and the probability of dyadic cooperation between BaYaka hunters and Yambe men who organise shotgun hunts. We found that BaYaka hunters’ attributes, but not Yambe attributes, predicted inter-group cooperation in shotgun hunting activities. These results provide novel insights into the mechanisms promoting inter-group cooperation in this setting, with implications for our understanding of how exchange based on specialised knowledge and skills can stabilise cooperation between groups more widely.

First, we found that those BaYaka hunters nominated as highly skilled by their peers were more likely to cooperate in shotgun hunting with Yambe, in support of H1. Prior work on men's reputations for hunting has shown that people in hunting communities track hunters’ abilities and may preferentially cooperate or associate with good hunters (Lew-Levy et al., 2021; E. A. Smith, 2004; K. M. Smith & Apicella, 2020), and that good hunting is associated with fitness-relevant variables (Apicella, 2014; Boyette et al., 2023; E. A. Smith, 2004; C. von Rueden et al., 2008). This is the first study we know of to demonstrate the impact of reputation for hunting on probability of inter-group cooperation. Here, it appears the Yambe also track BaYaka hunters’ reputations, and preferentially cooperate with the most-skilled hunters. The Yambe have much to gain from successful hunts, and they also stand to lose their investment of cash (on cartridges, batteries for a headlamp for night hunting and cigarettes for the hunter) should he fail to return with game. Thus, it makes sense that they pay attention to this highly relevant information about their available pool of hunters.

Additionally, though, this result may have implications for studies of cross-cultural competence in maintaining cultural diversity (Bunce, 2021). For instance, Bunce has shown that minority cultures can persist in multi-cultural environments with uneven power when individuals from the minority culture are competent in (i.e. maintain knowledge of) the majority culture. While these models address norms specifically, awareness of individual variation in critical group-specific skills or knowledge within other cultural groups (e.g. hunting skill, traditional ecological knowledge) may be another dimension of cross-cultural competence worth investigating as a means of preserving cultural diversity in contexts of exchange. That is, when one group depends on the knowledge of members of another group for their own economic benefit or survival in times of need, they should attend to and value not only the norms of another group, which govern interaction, but also the distribution of specific types of skill or knowledge in that group, which would govern the relative gains from interaction with any particular individual in that group. Anecdotally, in our experience in the study village and others nearby, farmers recognise and value the diversity of skills among BaYaka, not only for hunting but also for traditional medicinal knowledge, musical aptitude and more. It is also worth noting that while the Yambe are numerically the minority in the study village, they share norms with other groups in the region to a greater degree than do the BaYaka. Combining the Yambe with this larger population of cultural groups with overlapping norms (i.e. the Bantu-speaking farmers) suggests that majority group knowledge of minority groups can also be important if the minority possesses skills or knowledge valued by the majority, in contrast to the case of norms as explored by Bunce (2021).

Our other results show that BaYaka hunters are more likely to cooperate in shotgun hunting when they have more children, as we predicted (H2). Meat is a key food in BaYaka culture as well as the main source of animal protein (J. Lewis, 2008), and ‘meat hunger’ has been identified as a cause of decreases in well-being in this region and other parts of the Congo Basin (Doremus, 2019; Dounias & Ichikawa, 2017). Prior research has also specifically demonstrated that finding food, especially meat, and sharing it with their families is a crucial valued role for BaYaka fathers (Boyette et al., 2020). Furthermore, a sample of BaYaka hunters in the study village also unanimously said that they hunted for the Yambe with shotguns because it gives them a chance to provide meat to their families using a preferred hunting weapon (Kandza et al., 2023). These new results confirm their claims, and provide a more nuanced view into how individual participation in inter-group resource exchange – here, exchanging the means of production for shares of the resources produced – can be integral to family economies. As such, these results also point to pathways through which men's parental investment strategies motivate cooperation outside the family (Gettler, 2016), including across cultural boundaries.

Contrary to our predictions, we did not find considerable effects of any Yambe attributes on cooperative relationships in shotgun hunting. We expected that several factors might matter, including Yambe status, which might attract more cooperative partners, natal and marital relationships, because these would increase the number of partners possible from among fictive kin, and forms of wealth, which could increase the opportunities to arrange hunts. However, it seems that these factors did not influence the likelihood of any particular ties. Rather, probably because of overlapping kinship/fictive kinship networks, every Yambe has relatively equal likelihood of hiring those BaYaka who are the best hunters or who are seeking to hunt (e.g. those with greater need or motivation) – that is, most Yambe can claim rights to cooperate with many hunters, who tend not to refuse. The lack of any effect of Yambe status or wealth on partner choice also reflects how integrated shotgun hunting is into the local Yambe economy, such that it is one of many activities that everyone does. Even though higher status or wealthier people may have more access to resources, which they could potentially use to buy cartridges, it may be the BaYaka who get to decide, and they do not preferentially choose to work for higher status or wealthier Yambe, consistent with their own interest only in the meat gained from the hunt, and with their indifference to Yambe status norms. To confirm such speculation, future research might examine BaYaka preferences for specific attributes in their cooperative partners, such as respect for autonomy or generosity, which are valued traits within BaYaka culture (Boyette & Lew-Levy, 2019).

While not predicted, our results also provide evidence that a hunter's age is also probably associated with cooperation (Figure 2). Although our models could not entirely rule out no effect, the suggested effect is worth discussing. Prior work on inter-group cooperation in similar mixed-subsistence populations found that people were more likely to cooperate with out-group strangers if they had more familiarity with other cultures and if they perceived people in an out-group to be ‘good’ (Pisor & Gurven, 2018). What is especially noteworthy here is that most of our sample of BaYaka and Yambe men have known each other and interacted throughout much of each other's lives, but only BaYaka age predicted cooperation, not Yambe age. There are at least two non-mutually exclusive explanations for this result based on the dyadic nature of cooperation as examined here. One is that the Yambe tend to recruit older hunters, independent of their skill. This may be because, while reputation as a skilled hunter was clearly a more reliable predictor of cooperation in our models, age may indicate greater reliability in general, as hunting skills tend to increase generally with age until relatively late in adulthood (Koster et al., 2020). A second explanation is that since it appears that BaYaka have relatively greater control over partner choice, perhaps they increase their number of Yambe partners across their lifetime. This explanation may be more consistent with our other, negative finding – that no Yambe attributes predicted cooperation in our models.

Taking a wider view, these results may also have implications for our understanding of the balance of power (Cochran & O'Connor, 2019; Ensminger & Knight, 1997) in forager–farmer exchange relationships across space and time. As is common in other contemporary forager–farmer exchange contexts, BaYaka are often characterised as the disempowered group, and often experience discrimination in farmer spaces (Woodburn, 1997). However, our data are consistent with ethnographic studies (Köhler & Lewis, 2001; J. Lewis, 2005) that demonstrate that the BaYaka are also actively pursuing their own strategies in those contexts in which they have autonomous control – in other words, when the Bantu depend on their skills and knowledge of the forest (for other groups see, e.g. Blackburn, 1982; Fortier, 2001). Here, cooperation in shotgun hunting is driven by the needs of BaYaka men's families and by their individual skill as hunters, not by Yambe needs or their social influence over their BaYaka fictive kin. Hunting skill is crucially dependent on knowledge and experience in the forest. Thus, the maintenance of the forest and of BaYaka economic power vis-à-vis the farmers and others are linked. In general, then, we might expect forager persistence wherever they are able to maintain exclusive access to knowledge and skill acquired through experiential learning in their home ecologies.

Furthermore, our results may have implications for our understanding of the role of hunting in human cooperative behaviour. In our prior study, we report that Yambe always proposed the hunts (Kandza et al. 2023), thus we have confidence it was the Yambe choosing the hunters, and not the hunters choosing to cooperate because it would demonstrate their skill. As such, our results support the view that men's hunting – in this case via this mechanism of intercultural exchange – is primarily a means of provisioning (Marlowe, 2003; Wood & Marlowe, 2013, 2014), and not a means of signalling other qualities to observers (Hawkes, 1991; Hawkes & Bliege Bird, 2002). However, we cannot rule out the possibility that good hunters gain additional benefits from others’ knowledge of their skills, such as favoured treatment by the Yambe (e.g. larger shares of palm wine, reciprocity in other cooperative domains) or social support by other BaYaka (Boyette et al., 2023).

Several of our measures were relatively coarse-grained, and thus we acknowledge this limitation on the inferences that we can make. Indeed, the data did not strongly predict the posterior distribution, indicating relatively weak predictive power of the model based on our data (Figure S2). This information can guide future studies in using more detailed measures. For example, in terms of the effects of Yambe wealth, there may still be direct effects that we did not capture with the measures used here. Wealthier Yambe should be able to buy more cartridges and other supplies to organise more hunts, which should lead to more cooperation. Alternatively, wealthier people may diversify their investments, and thus may not pursue proportionally more hunting, but may cooperate more across different types of exchange (e.g. collection and sale of palm oil, palm wine, or *Gnetum* leaves). Captured by the U in our DAG (Figure 1), there are also probably other unmeasured Yambe traits that could impact their interests in shotgun hunting directly or indirectly, through their effects on individuals’ wealth, status or fertility. Future work will have to focus on these additional dimensions of inter-group cooperation in this setting. We also acknowledge that our measure of cooperation only reflects whether or not a particular Yambe and particular BaYaka had cooperated, not, for instance, how frequently. Yambe attributes such as wealth and status may indeed play more of a role in the frequency with which they organise hunts vs. the diversity of their collaborators. This is another dimension of this inter-group cooperative system worth investigating in future research.

## Conclusion

To summarise, our study indicates that hunters’ level of skill and their number of children both predicted cooperation between BaYaka hunters and Yambe. Yambe attributes, in contrast, had no effect on variation in cooperation, probably because of overlapping fictive kinship networks that allow Yambe access to a range of hunters and because of low variation among the Yambe in participation in shotgun hunting exchanges. These findings advance our understanding of the impact of individual-level variation on the stability of inter-group cooperation, and on the importance of tracking reputations across cultural boundaries. Additionally, though, these findings have implications for biodiversity conservation in the study region.

The shotgun is the preferred hunting method in the region because of its efficiency (Kandza et al., 2023). While the BaYaka participate in shotgun hunting to provision their families, the Yambe and other farming peoples in the region earn cash through selling hunted meat on the bushmeat market (Duda et al., 2017; Kandza et al., 2023). This market is only growing in correspondence with the influx of cash and wage-labour jobs to the region driven by the timber industry, whose road construction activities also make it easier to transport bushmeat out of the region (Poulsen et al., 2009; Wilkie et al., 2000). The BaYaka and the non-BaYaka groups with whom they have ties have long been integrated into global markets for forest products, including duiker pelts, ivory, rubber and cash crops (Bahuchet & Guillaume, 1982; J. Lewis, 2005). However, in the context of the dramatic loss of biodiversity across the planet (Pecl et al., 2017) and the range of local impacts of defaunation owing to hunting (Fa & Brown, 2009; Ichikawa et al., 2017; Poulsen et al., 2009; Remis & Jost Robinson, 2012), the current situation is certain to test the resiliency of both the forest and the people who depend upon it. Inter-group cooperation has probably been central to adaptation to ecological, economic and social changes in the Congo Basin throughout the history of human settlement in this region (Boyette et al., 2022). Research on these dynamics will remain important as the BaYaka, the Yambe and others continue to adapt to the hastened pace and scale of anthropogenic change.

## Supporting information

Kandza et al. supplementary material Kandza et al. supplementary material

## Supplementary material

To view supplementary material for this article, please visit https://doi.org/10.1017/ehs.2024.14

## Research transparency and reproducibility

The data used in this study is available in the following GitHub repository: https://github.com/haneuljangkr/shotgun-hunting-inter-group-cooperation
