# Supplementary material for: Dyadic inter-group cooperation in shotgun hunting activities in a Congo Basin village
Source: Evol Hum Sci. 2024 Apr 1;6:e22. doi: 10.1017/ehs.2024.14 (PMC11058523; doi:10.1017/ehs.2024.14)
Supplement: Kandza et al. supplementary material [file S2513843X24000148sup001.docx]

**Electronic Supplementary Information**

Table S1. Questions asked to BaYaka hunters and Yambe gun owners.

| **Questions Asked to BaYaka hunters** |
| --- |
| How old are you? |
| How many children did you have? |
| What is your father’s name**?** |
| What is your mother’s name**?** |
| Who are the best hunters that you know? |
| **Questions asked to Yambe gun owners** |
| How old are you? |
| How many children did you have? |
| What is your father’s name**?** |
| What is your mother’s name**?** |
| Where were you born? |
| Where was your wife born? |
| Where was your mother born? |
| Does your house have a metal roof? |
| Are you one of the village council members? |
| Which BaYaka hunters do you usually hire to do shotgun hunting? |

Table S2. Results of model comparison. Models with covariates were compared with the base line model by using "loo2" (method loo_model_weights in brms package) to obtain weights. The models with covariates were generally more favored by model comparison than the baseline model, except the Yambe model including Yambe’s age and mother’s natal location.

| **Base line model** | **1-BaYaka skills** |
| --- | --- |
| 0.248 | 0.752 |
| **Base line model** | **2-BaYaka need** |
| 0 | 1 |
| **Base line model** | **3-Yambe need, status, and wealth** |
| 0.369 | 0.631 |
| **Base line model** | **4-Yambe maternal inheritance of ties** |
| 1 | 0 |
| **Base line model** | **5-Yambe marriage inheritance of ties** |
| 0.44 | 0.56 |

******

Figure S1. Forest plots for random effects of Yambe (YA) and BaYaka (BY) IDs. These two plots show estimated differences in probabilities of shotgun hunting cooperation between each Yambe-BaYaka dyad. The point estimate is the median. The outer and inner lines represent 50% and 89% credibility intervals, respectively.


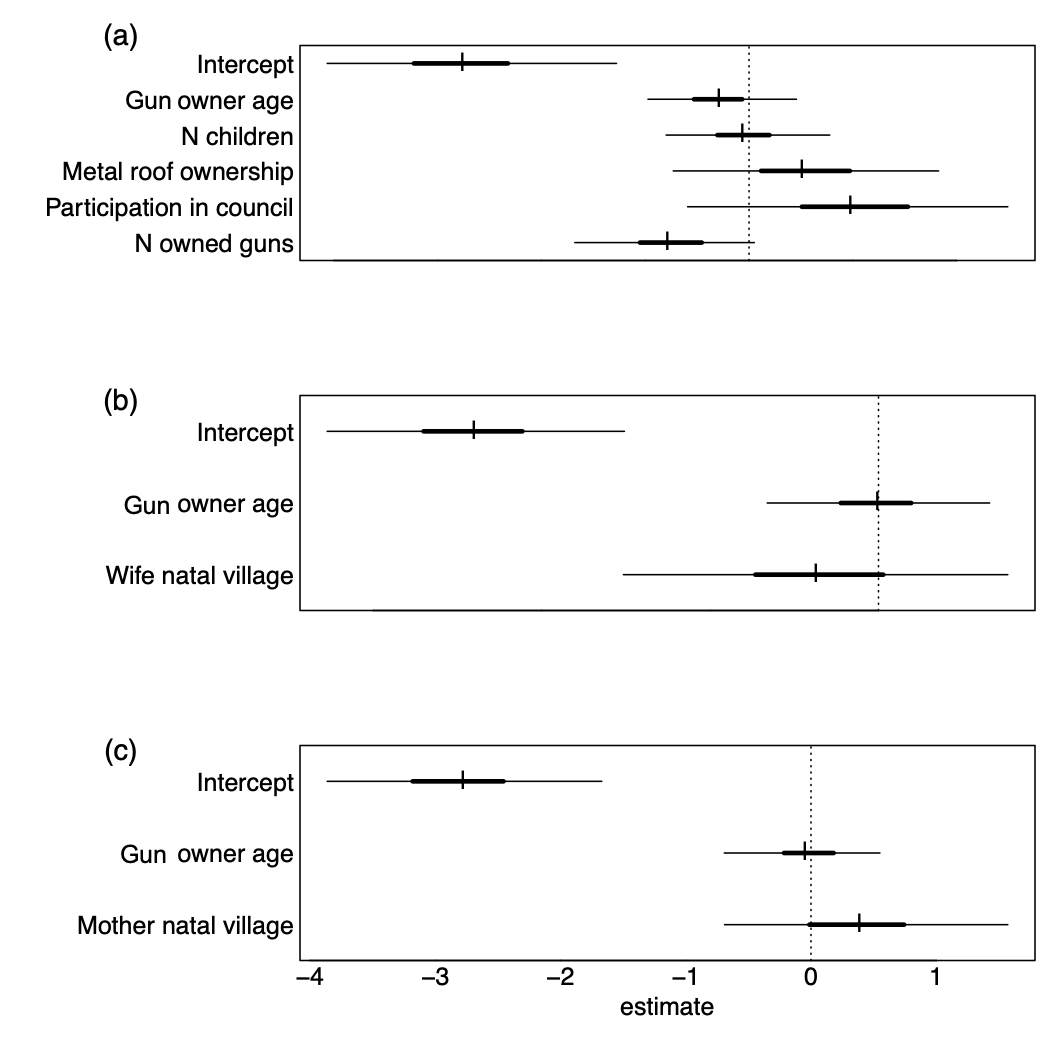


Figure S2. Forest plots for Yambe attributes. These three plots show estimated changes in probabilities of Yambe gun owners to participate in cooperative hunting with BaYaka hunters, depending on (a) the number of living biological children of the gun owners, metal roof ownership (yes/no), participation in council (yes/no) and the number of owned guns, (b) whether gun-owner's wife was born in the study village and (c) whether gun-owner's mother was born in the study village, accounting for hunter’s age. The point estimate is the median. The outer and inner lines represent 50% and 89% credibility intervals, respectively.

Figure S3. Density plot of prior (black dashed line) and posterior (orange solid line) distributions for parameters Yambe model with (Yambe age, the number of Children, metal roof presence, council participation and the number of guns) when the model was fit with weakly informative priors for all parameters. This plot shows how much the model updated upon seeing the data.
